# A Small RNA, SaaS, Promotes Salmonella Pathogenicity by Regulating Invasion, Intracellular Growth, and Virulence Factors

**DOI:** 10.1128/spectrum.02938-22

**Published:** 2023-01-23

**Authors:** Lin-lin Cai, Yun-ting Xie, Hai-jing Hu, Xing-lian Xu, Hu-hu Wang, Guang-hong Zhou

**Affiliations:** a Jiangsu Collaborative Innovation Center of Meat Production and Processing, Quality and Safety Control, Nanjing Agricultural University, Nanjing, People’s Republic of China; University of Maryland School of Pharmacy

**Keywords:** *Salmonella* Enteritidis, sRNA, SaaS, virulence, bacterial attachment, intracellular growth

## Abstract

Salmonella enterica serovar Enteritidis is a common foodborne pathogen that infects both humans and animals. The S. Enteritidis virulence regulation network remains largely incomplete, and knowledge regarding the specific virulence phenotype of small RNAs (sRNAs) is limited. Here, we investigated the role of a previously identified sRNA, Salmonella adhesive-associated sRNA (SaaS), in the virulence phenotype of S. Enteritidis by constructing mutant (Δ*saaS*) and complemented (Δ*saaS*/p*saaS*) strains. SaaS did not affect S. Enteritidis; it was activated in the simulated intestinal environment (SIE), regulating the expression of virulence target genes. We discovered that it directly binds *ssaV* mRNA. Caco-2 and RAW 264.7 cell assays revealed that SaaS promoted S. Enteritidis invasion and damage to epithelial cells while suppressing macrophage overgrowth and destruction. Furthermore, a BALB/c mouse model demonstrated that the deletion of SaaS significantly reduced mortality and attenuated the deterioration of pathophysiology, bacterial dissemination into systemic circulation, and systemic inflammation. Our findings indicate that SaaS is required for S. Enteritidis virulence and further highlight its biological role in bacterial pathogenesis.

**IMPORTANCE** Salmonella is a zoonotic pathogen with high virulence worldwide, and sRNAs have recently been discovered to play important roles. We explored the biological characteristics of the sRNA SaaS and developed two cell infection models and a mouse infection model. SaaS is an SIE-responsive sRNA that regulates the expression of virulence-targeted genes. Additionally, it differentially mediates invasion and intracellular growth for survival and infection of the epithelium and macrophages. We further found that SaaS enhanced bacterial virulence by promoting lethality, colonization, and inflammatory response. These findings provide a better understanding of the critical role of sRNA in bacterial virulence.

## INTRODUCTION

Salmonella is responsible for a wide range of foodborne diseases from mild, self-limiting gastroenteritis to life-threatening systemic infection, which has been reported to cause approximately 180 million cases of diarrhea annually and accounts for 41% of all diarrhea-associated deaths (1). The Salmonella genus contains over than 2,600 serotypes, with Salmonella enterica serovar Typhimurium and Salmonella enterica serovar Enteritidis being the most common isolates (2). S. Enteritidis, a facultative intracellular Gram-negative pathogen, can break through the epithelial cell layer of the intestine, replicate in phagocytes, disseminate, and eventually cause systemic infection, depending on various virulence factors that play synergistic roles in its pathogenic process.

Hundreds of putative virulence factors associated with S. Enteritidis virulence have been identified thus far. Major virulence factors are classified into three categories based on their sources and biological functions in the bacterial life cycles: genes within Salmonella pathogenicity islands (SPIs), virulence plasmids such as *spvABCD*, and flagella and pili, such as *motB*, *fliC*, *csgA*, and *csgD*. SPIs are the most essential and extensively investigated, with SPI-1 and SPI-2 being the best studied. SPI-1 and its encoding type III secretion system 1 (T3SS-1) are mainly invasion genes, such as *hilA*, *hilD*, *invA*, and *prgJ*, whereas SPI-2 and its T3SS-2 contain genes required for intracellular survival and replication, such as *spiA*, the *ssa* operon encoding structural components, and the *sse* operon encoding translin and effector proteins. These T3SSs are specialized organelles that deliver effector proteins to the cytosol of the host cells (3). Highly regulated expression of SPI genes and those encoding their effector proteins has been observed both *in vitro* and *in vivo* and is required for bacterial pathogenesis and infection (4, 5).

Noncoding small RNAs (sRNAs), a class of major posttranscriptional regulators, have recently been discovered and identified to regulate the expression of abovementioned virulence factors, particularly those in SPIs. Bacterial sRNAs range in length from 50 to 500 nucleotides and are typically untranslated, consistent with endogenous noncoding microRNAs (miRNAs) found in plants and animals (6). Although the first evidence for the existence of bacterial sRNA was reported in 1975 (7), the majority of bacterial sRNA discoveries have occurred over the past 20 years. *trans*-encoded mRNAs are the primary regulatory objects of sRNAs in comparison to proteins, and their translation is modulated by imperfect base pairing. Much pioneering work on sRNA-regulated regions has been done, with the 5′ untranslated region, including the Shine-Dalgarno sequence and the start codon, being shown to be the most essential sequence for base pairing, followed by the coding sequence of the mRNAs (8–10). They have mostly shown significant effects on various cellular processes in bacteria *in vitro* (11), particularly sensing and responding to stress conditions, such as DsrA in oxidative stress (12), CyaR in multiple chemical stresses (13), and RyhB in acidic environments (14).

Following ingestion, Salmonella is exposed to diverse host environments and stresses, including gastric acid, anaerobic conditions, gut microbes, and immune defense mechanisms of phagocytic or nonphagocytic cells. Rapid perception and high adaptability are required for survival; even though sRNAs are considered to have the potential to function and perform effectively, only a small subset of sRNAs have been elucidated to regulate virulence in hundreds of Salmonella candidates. The majority of them are from *S*. Typhimurium, such as PinT, IsrJ, IstR, and SroA, which contributes to *phoP*-mediated regulation of SPI-1 T3SS (15), controls the production of effector proteins (11), inhibits growth allowing DNA repair, and putatively imports thiamine and thiamine pyrophosphate (16), respectively. Several sRNAs, including STnc640 and RyhBs, which contribute to virulence in S. Enteritidis have been recently discovered (17, 18). The investigation of sRNA in S. Enteritidis mostly focuses on the regulation of virulence gene expression, while research on the exact virulence phenotype in bacterial pathogenesis caused by sRNA, which is crucial for analyzing the pathogenesis of S. Enteritidis, is still limited and fragmentary.

Our earlier research revealed that the Salmonella adhesive-associated sRNA (SaaS) is associated with S. Enteritidis biofilm formation (19), which is closely associated with colonization. This indicates a potential correlation between SaaS and S. Enteritidis virulence. However, the biological functions of SaaS in S. Enteritidis pathogenicity remain unelucidated. In this study, we aimed to determine the biological role of SaaS in S. Enteritidis pathogenicity as well as its contribution to bacterial virulence. This paper improves knowledge of the virulence regulatory network of S. Enteritidis, emphasizes the role of sRNA in pathogenicity, and provides an experimental basis for target of Salmonella control.

## RESULTS

### SaaS is a SIE-responsive sRNA that regulates virulence gene expression and directly interacts with *ssaV*.

**FIG 1:**
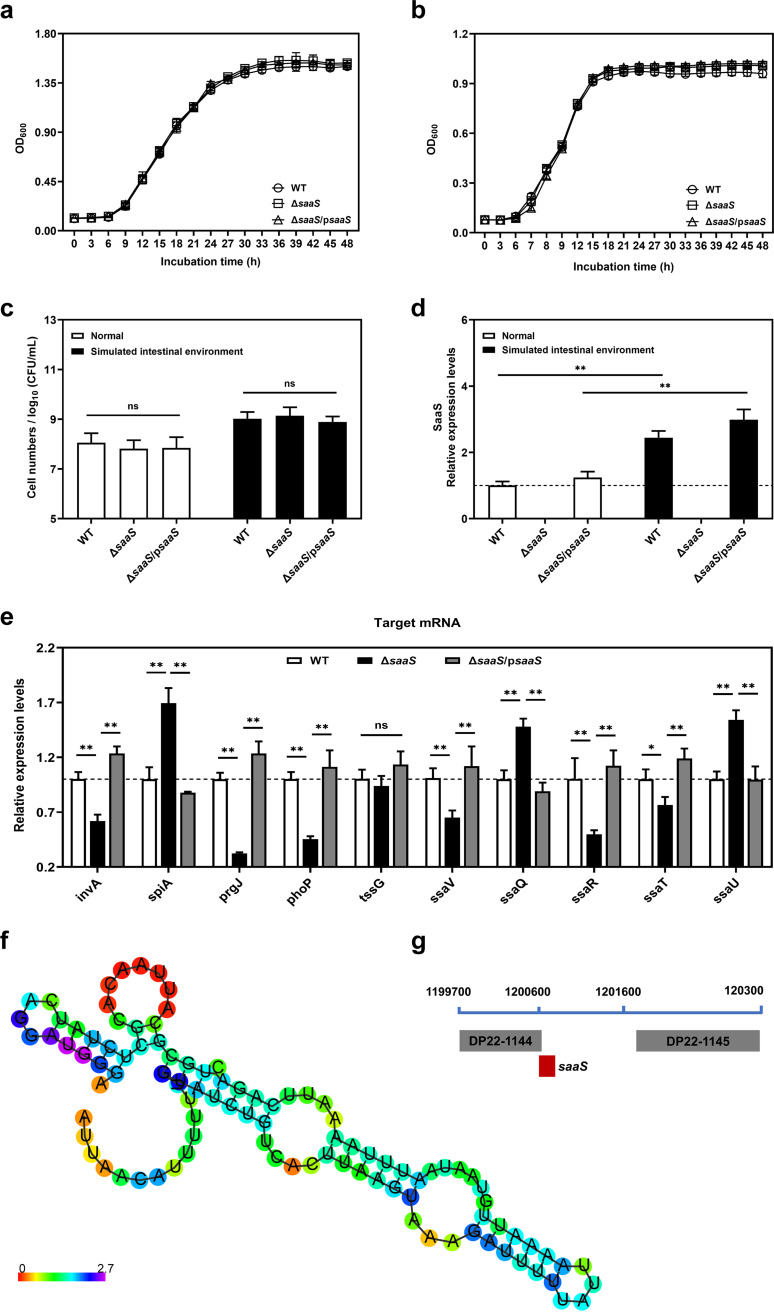
Growth characteristics and expression levels of SaaS and virulence target genes in S. Enteritidis. (a) Growth curves in LB broth. (b) Growth curves in the SIE. (c) Bacterial count in LB broth and the SIE after 12 h of incubation. (d) Expression of *saaS* gene in LB broth and SIE compared with that of WT in LB broth set as 1. (e) Expression of virulence target genes of Δ*saaS* mutant compared with those of WT under the SIE. (f) Predicted secondary structure of SaaS using RNAfold. The structure is colored by base-pairing probabilities. For unpaired regions the color denotes the probability of being unpaired. (g) Location information of *saaS* in the S. Enteritidis genome. SIE, simulated intestinal environment. Data are represented as means ± SD. In each independent experiment, there were at least five replicates per group. Statistical significance was determined against the same strain for panel c or the Δ*saaS* group for panels c and e using Student’s *t* test. *, *P* < 0.05; **, *P* < 0.01; ns, not significant.

We investigated the effect of SaaS sRNA on Salmonella bacterial proliferation. The wild-type (WT) and Δ*saaS*/pSaaS groups showed similar changes in this assay. In the presence or absence of a simulated intestinal environment (SIE), the growth patterns of WT and Δ*saaS* strains were similar (Fig. 1a and b), and there was no significant difference (*P* > 0.05) in the viable cell counts of the three strains (Fig. 1c), indicating that SaaS was not directly involved in S. Enteritidis growth. The expression of *saaS* was increased by 2- to 3-fold in the SIE compared to that in normal LB broth, regardless of whether it was for the WT or Δ*saaS*/p*saaS* strain (Fig. 1d), which means the sRNA SaaS could be activated by the intestine environment.

**FIG 2:**
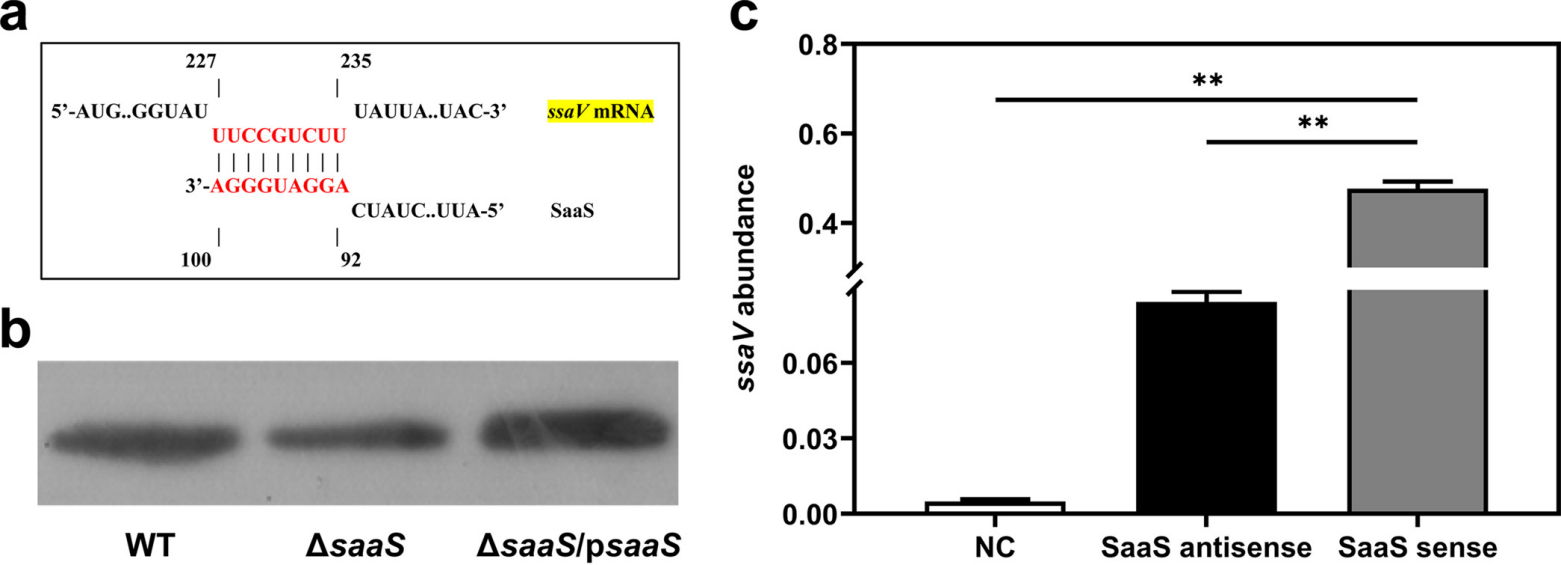
Correlation between SaaS and *ssaV*. (a) Predicted SaaS and *ssaV* mRNA interaction using IntaRNA. (b) Protein levels of SsaV in WT, Δ*saaS*, and Δ*saaS*/p*saaS* strains under simulated intestinal environment by Western blotting. (c) Expression of *ssaV* mRNA detected by RT-qPCR in biotin pulldown assay. NC, negative control. Data are represented as means ± SD. Statistical significance was determined against NC and SaaS antisense using Student’s *t* test. *, *P* < 0.05; **, *P* < 0.01.

The interaction of sRNA with its mRNA targets is essential for direct sRNA regulation. Under SIE, the virulence-associated target mRNA levels of *invA*, *prgJ*, *phoP*, *ssaV*, *ssaR*, and *ssaT* were significantly (*P* < 0.05) lower in the Δ*saaS* strain than in the WT (Fig. 1e). In contrast, the mRNA expression levels were significantly (*P* < 0.05) higher in the Δ*saaS* strain than in the WT for the other three genes, *spiA*, *ssaQ*, and *ssaU*. This indicates a diverse regulatory framework for SaaS. The secondary structure predicted by RNAfold and the location of *saaS* in the genome are shown in Fig. 1f and g. We further explored the interplay between SaaS and *ssaV* mRNA. As shown in Fig. 2, the protein levels of SsaV were significantly decreased in the Δ*saaS* strain compared to the WT, which was consistent with the change in mRNA levels. The pulldown assay was applied to make sure the direct interaction existed between SaaS and *ssaV*. As expected, the *ssaV* expression was markedly enriched by SaaS pulldown, which confirmed the direct combination. Taken together, we can conclude that the sRNA SaaS is not directly required for S. Enteritidis growth but regulates the expression of virulence genes under SIE.

### sRNA SaaS promotes intestinal barrier dysfunction and bacterial immune escape.

**FIG 3:**
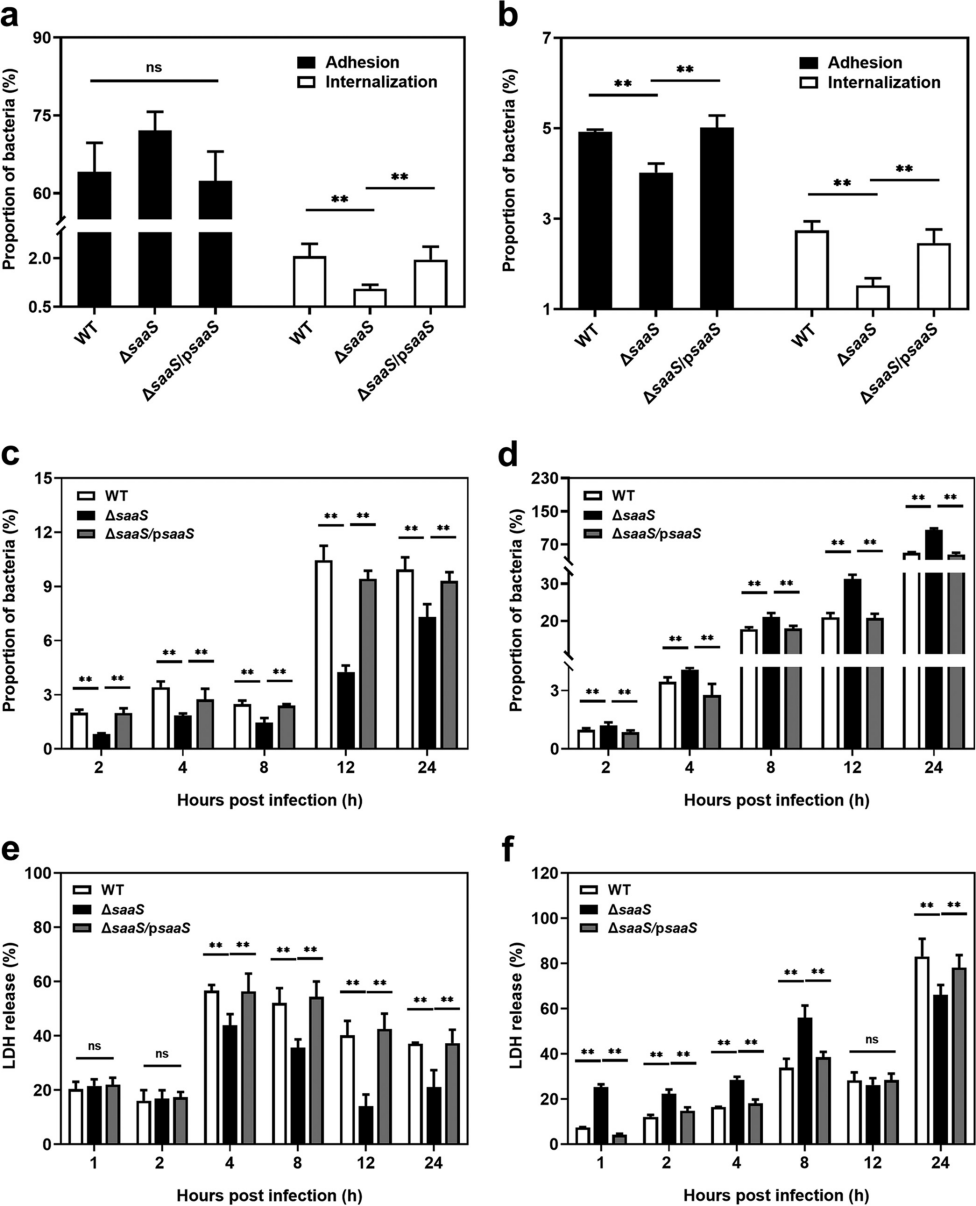
The involvement of SaaS in invasion and damage of S. Enteritidis to host cells. (a and b) Adhesion and internalization of S. Enteritidis to Caco-2 cells. (b) Adhesion and internalization of S. Enteritidis to RAW 264.7 cells. (c) Intracellular replication of S. Enteritidis in Caco-2 cells. (d) Intracellular survival of S. Enteritidis in RAW 264.7 cells. (e) LDH release of Caco-2 cells treated with S. Enteritidis. (f) LDH release of RAW 264.7 cells treated with S. Enteritidis. Data are represented as means ± SD. In each independent experiment, there were at least eight replicates per group. Statistical significance was determined against the Δ*saaS* group using Student’s *t* test. *, *P* < 0.05; **, *P* < 0.01.

Passage through the intestinal epithelial cell barrier is a key step in the pathogenesis of S. Enteritidis. The influence of sRNA SaaS on the invasion and intracellular replication or survival of S. Enteritidis *in vitro* was investigated using Caco-2 and RAW 264.7 cell lines, respectively. There was no significant difference (*P* > 0.05) in the ability of the WT and Δ*saaS* strains to adhere to Caco-2 cells. In contrast, there was a marked reduction in adherence to RAW 264.7 cells and internalization of both cells for the Δ*saaS* strain compared with the WT (Fig. 3a and b). The internalization rates of the WT in Caco-2 and RAW 264.7 cells were approximately 2-fold higher than those of the Δ*saaS* strain. It is assumed that SaaS contributes to the internalization of S. Enteritidis into host cells. In Caco-2 cells, the WT demonstrated significantly higher levels of replication ability than the Δ*saaS* strain at all times (Fig. 3c); however, the Δ*saaS* strain had a higher proportion of viable bacterial counts in RAW 264.7 cells (Fig. 3d).

Cell cytotoxicity after SaaS invasion was evaluated based on lactate dehydrogenase (LDH) release. Except for the 1st and 2nd hours, the WT promoted the release of LDH. The LDH activities in all WT-infected Caco-2 cells were significantly greater (*P* < 0.05) than those in the Δ*saaS* group (Fig. 3e), which was consistent with the abovementioned results for intracellular replication. For RAW 264.7 cells, the change in LDH activities was opposite that of Caco-2 cells in all groups. LDH release in the WT group was lower (*P* < 0.05) than that in the Δ*saaS* group, indicating weakened damage to the cellular membranes during 8 h of infection (Fig. 3f). Notably, during infection in the following 16 h, LDH release was improved in WT-infected cells and was higher than that in the Δ*saaS* group.

### sRNA SaaS strengthens the virulence of S. Enteritidis *in vivo*.

**FIG 4:**
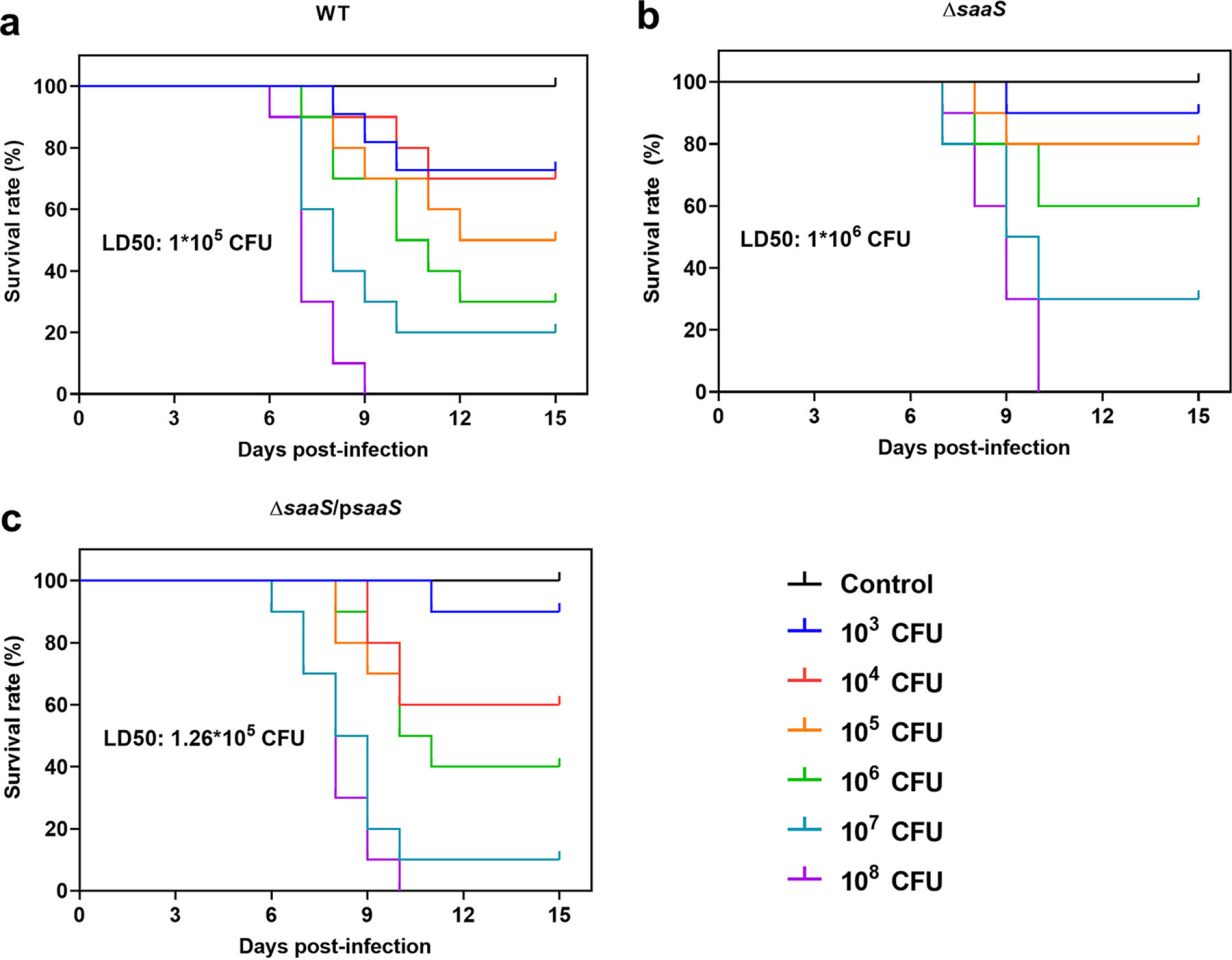
*In vivo* survival analysis of BALB/c mice injected with 10^3^ to 10^8^ CFU of S. Enteritidis and PBS. (a) WT groups; (b) Δ*saaS* groups; (c) Δ*saaS*/p*saaS* groups. In each independent experiment, there were 10 replicates per group.

SaaS activation significantly regulates the expression of virulence genes, invasion, and intracellular multiplication in the host cell, indicating the ability to regulate bacterial pathogenicity *in vivo*. Therefore, we subsequently explored the effect of SaaS on bacterial virulence *in vivo* in a mouse infection model. Compared with Δ*saaS* strain-infected mice, there was a quicker death and an approximately 16% lower survival rate in WT-infected mice (Fig. 4). At the end stage of infection, all mice developed typical clinical symptoms, such as shivering, rough coat hair, lethargy, and white secretions surrounding both eyes. Mice infected with the phosphate-buffered saline (PBS) control showed an overall survival rate of 100% at 14 days postinfection and no obvious symptoms. The 50% lethal dose (LD_50_) of the Δ*saaS* group was calculated to be almost 8- to 10-fold higher than that of the WT and Δ*saaS*/p*saaS* groups (1 × 10^5^, 1 × 10^6^, and 1.26 × 10^5^ CFU for the WT, Δ*saaS*, and Δ*saaS*/p*saaS* groups, respectively), which means that the deletion of SaaS attenuated the virulence of S. Enteritidis in mice.

### sRNA SaaS strengthens the deterioration of ethophysiology and bacterial dissemination.

**FIG 5:**
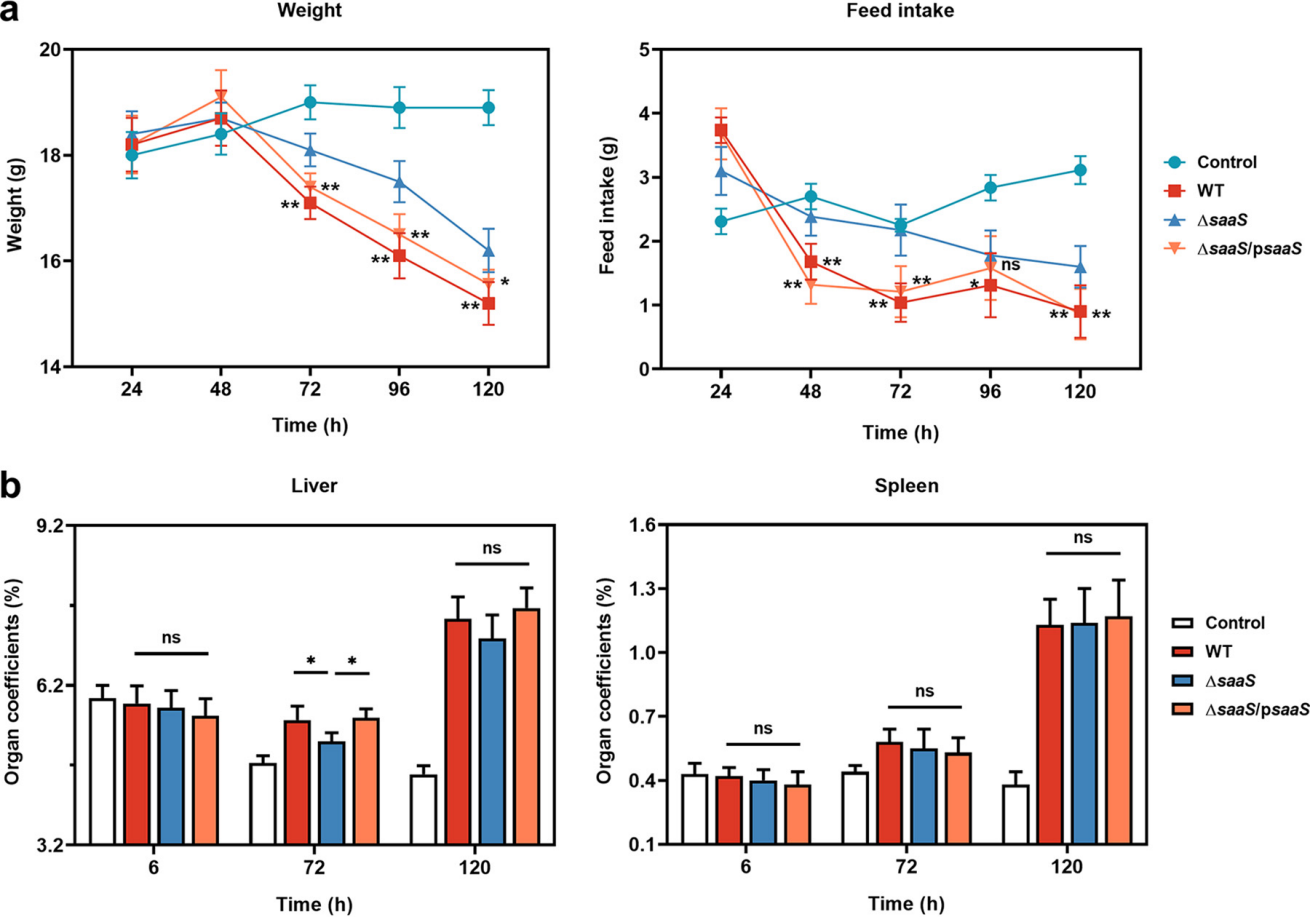
Ethophysiology of mice infected with S. Enteritidis. (a) Changes in weight gain and food intake evaluated every 24 h. (b) Organ coefficients of liver and spleen. Data are represented as means ± SD. In each independent experiment, there were at least eight replicates per group. Statistical significance was determined against the Δ*saaS* group using Student’s *t* test. *, *P* < 0.05; **, *P* < 0.01.

We investigated the effect of the sRNA SaaS on the systemic response *in vivo*. An acute inflammation model was constructed for evaluating the ethophysiology. Changes in body weight and feed intake were monitored every 24 h in all the groups. The body weight and feed intake decreased over time in all treatment groups. There was no significant difference in body weight during the first 48 h of infection or in feed intake during the first 24 h of infection (Fig. 5a and b), However, after this time period, both body weight and feed intake significantly decreased (*P* < 0.05) in the WT group. Simultaneously, the organ coefficients of the liver and spleen at specific time points (6, 72, and 120 h) were calculated. In contrast to the change in body weight and feed intake, the organ coefficients of both organs continued to increase in all treatment groups, whereas those of the control group declined (Fig. 5c and d). There was a significant difference (*P* < 0.05) in the liver at 72 h when the organ coefficient was higher in the WT group than in the Δ*saaS* group. No significant differences were observed in the organ coefficients of the spleen.

**FIG 6:**
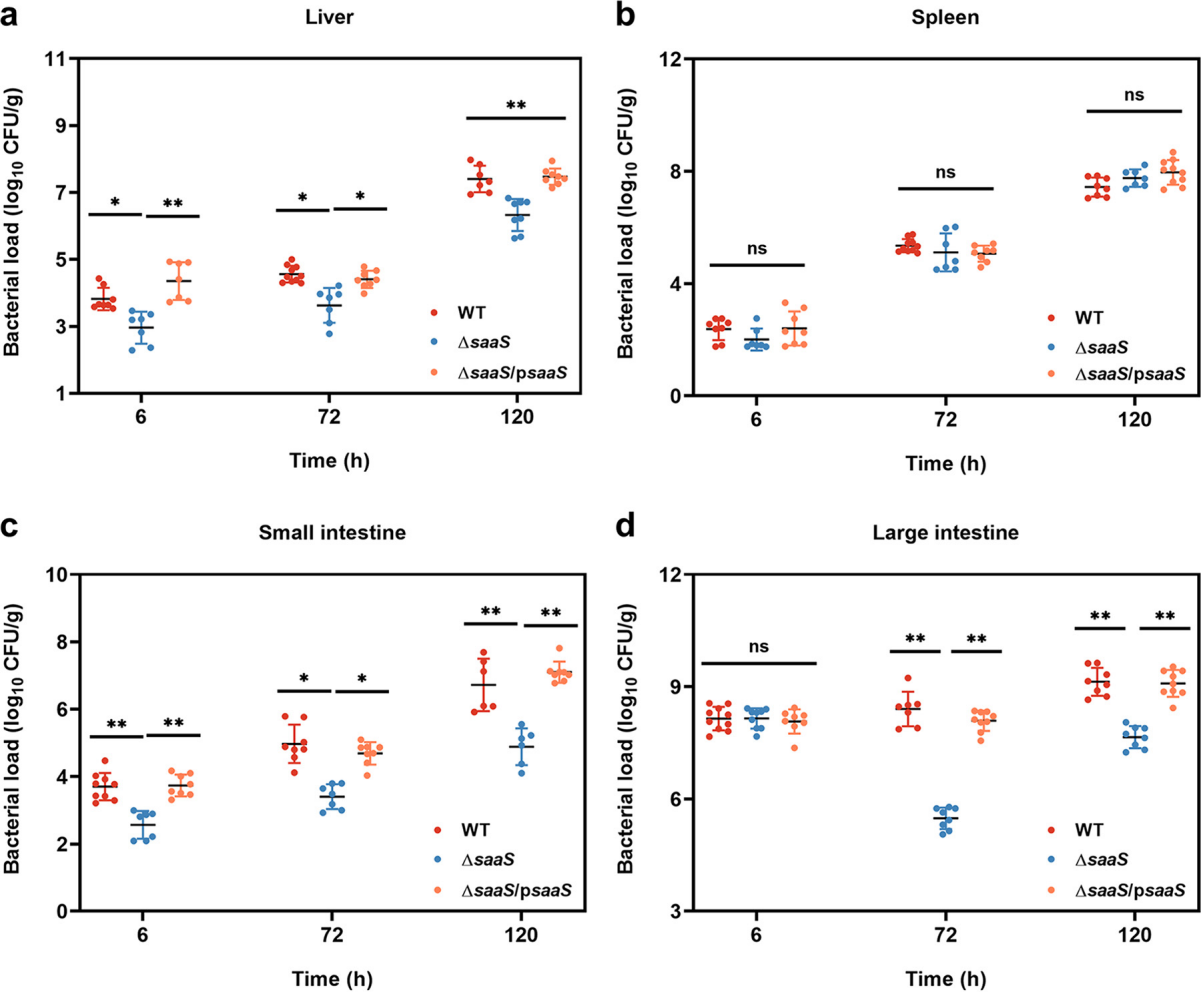
Bacterial dissemination of S. Enteritidis WT, Δ*saaS*, and Δ*saaS*/p*saaS* strains in various tissues of mice. (a) Bacterial numbers on liver. (b) Bacterial numbers on spleen. (c) Bacterial numbers on small intestine. (d) Bacterial numbers on large intestine. Data are represented as means ± SD. In each independent experiment, there were at least eight replicates per group. Statistical significance was determined against the Δ*saaS* group using Student’s *t* test. *, *P* < 0.05; **, *P* < 0.01.

Furthermore, the colonization efficiencies of WT, Δ*saaS*, and Δ*saaS*/p*saaS* strains in the tissues of BALB/c mice were compared. Overall, bacterial numbers in different organs increased during infection. With the exception of the large intestine at 6 h, the bacterial numbers of WT recovered from the liver (Fig. 6a), small intestine (Fig. 6c), and large intestine (Fig. 6d) were significantly higher than those of the mutant strain Δ*saaS* (*P* < 0.05), indicating that the SaaS deletion mutant increased microbial clearance in host tissues of the infected mice. No significant difference was observed in the spleen during the entire infection period (Fig. 6b), which was consistent with the organ coefficient results. These data confirm that SaaS is required for S. Enteritidis dissemination in systemic organs.

### sRNA SaaS strengthens the systemic inflammatory response.

**FIG 7:**
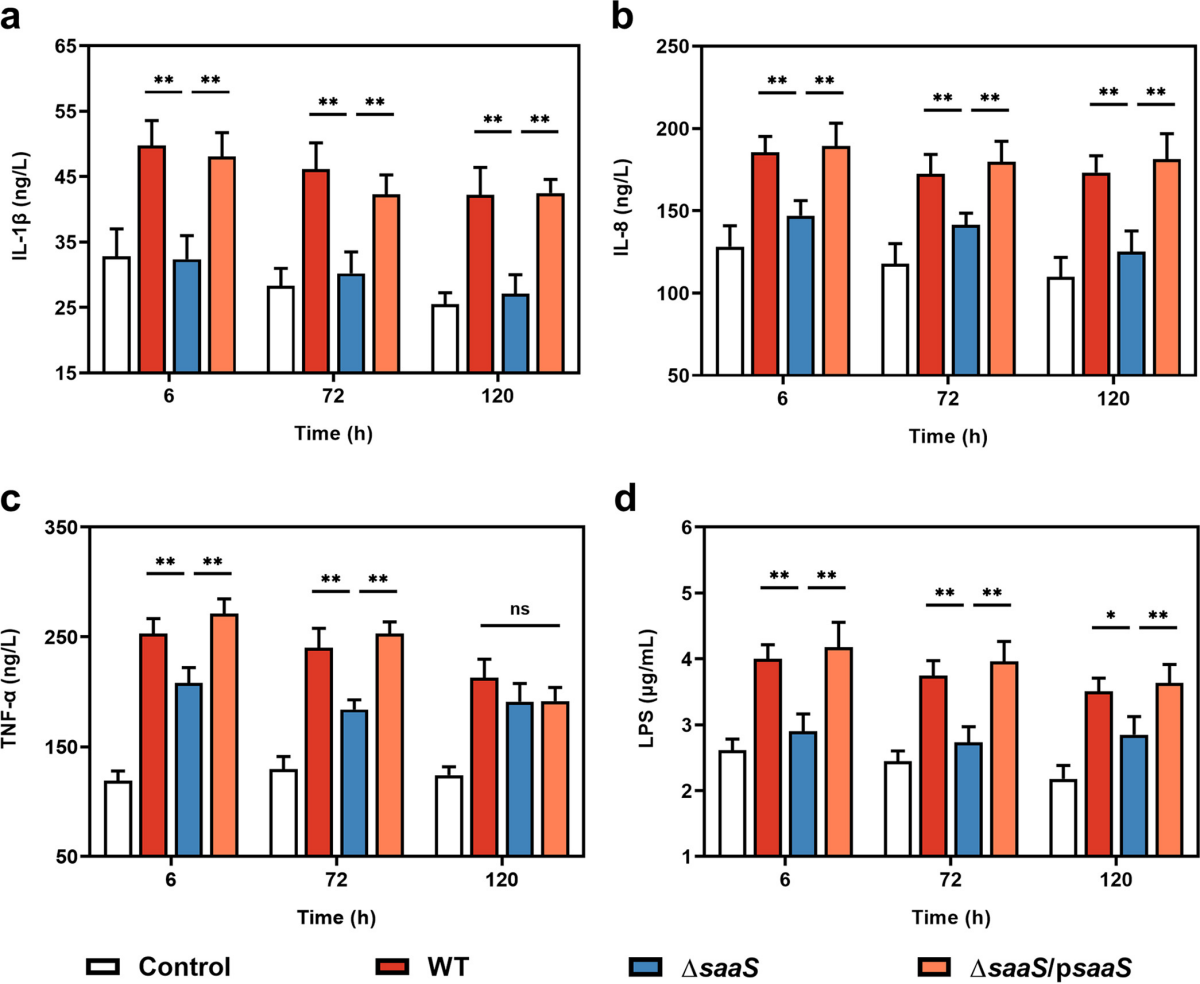
Inflammatory response and LPS concentration in serum of S. Enteritidis-infected mice. (a to c) Levels of inflammatory cytokines (IL-1β, IL-8, and TNF-α, respectively). (d) Levels of LPS. Data are represented as means ± SD. In each independent experiment, there were at least eight replicates per group. Statistical significance was determined against the Δ*saaS* group using Student’s *t* test. *, *P* < 0.05; **, *P* < 0.01.

Next, we used enzyme-linked immunosorbent assay (ELISA) to investigate whether SaaS mediated the secretion of cytokines *in vivo*, with the interleukin-1β (IL-1β), IL-8, and tumor necrosis factor alpha (TNF-α) levels in serum after infection being examined with ELISA. Lipopolysaccharide (LPS) levels, one of the factors responsible for cytokine release, were also measured (Fig. 7). At all time points, the serum concentrations of IL-1β, IL-8, and TNF-α were significantly higher in the WT-infected group than in the Δ*saaS* strain-infected group, except for TNF-α at 120 h. Notably, there was no significant difference in the levels of IL-1β and IL-8 between the PBS- and Δ*saaS* strain-infected groups during the entire infection. Similarly, the concentration of LPS was higher in the WT-infected group than in the Δ*saaS* strain-infected group at all time points. For Δ*saaS* strain-infected mice, the concentration of LPS increased only at the final 120-h time point. Thus, sRNA SaaS increased the concentration of LPS and induced proinflammatory cytokine release *in vivo* to trigger severe damage.

## DISCUSSION

S. Enteritidis is a common typical foodborne contaminant that infects both humans and animals, causing millions of foodborne diseases and deaths each year worldwide (20, 21). sRNAs have been demonstrated to be widely used by Salmonella to overcome adverse *in vitro* and *in vivo* environments and to play roles no less vital than those of transcription factors and nucleus-related proteins in the adaptation process and various physical activities. The role of the novel sRNA SaaS in Salmonella biological function was first evaluated in this study. Although not directly involved in the growth regulation of S. Enteritidis, the sRNA SaaS had significantly higher expression under SIE. Considering that SaaS is associated with biofilm formation (19), the promotion of *saaS* expression may be involved in the virulence of S. Enteritidis. As a result, the mRNA levels of virulence-associated target genes were examined in bacteria incubated in an SIE. In the absence of SaaS, the mRNA levels of *invA*, *prgJ*, *phoP*, *ssaV*, *ssaR*, and *ssaT* were downregulated, whereas those of *spiA*, *ssaQ*, and *ssaU* were upregulated. Among these genes, *invA* and *prgJ* belong to SPI-1, responsible for epithelial cell invasion, macrophage apoptosis, and phagocyte recruitment (22), while the rest, including *spiA* and the *ssa* operon (*ssaV*, *ssaQ*, *ssaR*, *ssaT*, and *ssaU*), belong to SPI-2 T3SS, which is required for intracellular survival and replication (23). Regulation of the type and mode of effector protein secretion is crucial for the latter. SsaV is the largest component of this injectisome, and its attenuation leads to a significant reduction in Salmonella virulence by decreasing the translocation of SPI-2 effectors (4). Furthermore, the pH of the host cell cytoplasm influences the SsaV-mediated control of the second secretion switch and secretion specificity (24, 25). Given the altered intestinal pH environment, the positive and direct interaction between the SaaS and *ssaV* activation models plays an essential role in Salmonella pathogenesis. For all of these reasons, we questioned how SaaS is involved in Salmonella pathogenicity, for example, the interaction with host cells including the invasion of epithelium and intracellular replication in phagocytes.

Effective dissemination of enteric pathogens requires a dysfunctional intestinal barrier. As a part of the mechanical barrier, epithelial cells play a key role in preventing localized or systemic infection by nontyphoidal Salmonella (26, 27). The Caco-2 cell line, an epithelial cell line, was chosen to preliminarily evaluate the contribution of SaaS to the intestinal barrier. Salmonella with a deletion of SaaS was defective in invasion and intracellular replication and caused less damage to the cell membrane of Caco-2 cells, which could be corrected by introducing a complementation construct containing the full-length SaaS sequence. From the host’s perspective, the greater the release in WT, the greater the loss of cell membrane integrity, which is attributed to cell necrosis, apoptosis, or autophagy during infection (28, 29). Salmonella can break through the cell membrane by mediating cytoskeletal rearrangements and changes in phosphoinositide dynamics, such as the cholesterol accumulation, Rab10 inhibition, and increased caveolin-1 levels at bacterial entry sites and cellular cholesterol redistribution (30–32). These findings could be explained in part by the upregulated mRNA expression of both *invA* and *prgJ* in the WT. In Salmonella, InvA is the major invasion protein that determines its ability to enter the epithelial cell, while *prgJ* serves for the passage of proteins that travel the T3SS. As a result, the sRNA SaaS was considered to contribute to intestinal physical barrier dysfunction by interacting with the target mRNAs *invA* and *prgJ* in S. Enteritidis infection. Further research into the mechanisms of sRNA SaaS interaction with these target genes in S. Enteritidis would be beneficial.

Following the destruction of the intestinal barrier and prior to colonization of other organs, successful systemic dissemination of the bacteria via the bloodstream requires the facilitation of migration of the infected phagocytes or predominantly macrophages (33). The behavior pattern of Salmonella mediated by SaaS in RAW 264.7 cells was found to be significantly different from that in Caco-2 cells. The different membrane structures of the two cell types may affect Salmonella adhesion mediated by SaaS, while a possible mechanism of immune evasion within phagocytic cells triggered by SaaS may be the cause of the contradictory replication and damage. Reduced intracellular replication of Salmonella suppresses antigen presentation and regulates the CD8 T cell response, while the overgrowth phenotype of Salmonella mutants displayed attenuated virulence within the phagocyte (34–36). Unlike epithelial cells, which are fixed as a part of the intestine, phagocytes are flexible and can be utilized by Salmonella to spread in the blood or organs (37). Mediated by SaaS, S. Enteritidis may prolong the life span of infected cells, increase the number of infected macrophages in the blood, and promote the spread of bacteria to host tissues. After reaching specific organs, after 12 h of infection in this study, macrophages may be lysed, and bacteria are released for further colonization. From a broad perspective of similar researches, SaaS has been the unique sRNA that promotes the ability of bacterial immune evasion for further infection. The expressions of *ssaV*, *ssaR*, and *ssaT* were upregulated while those of *spiA*, *ssaQ*, and *ssaU* were downregulated, indicative of a potential complex regulatory mechanism of SaaS for immune evasion in S. Enteritidis.

Considering that the interaction between bacteria and the host is very complex due to diverse host background conditions, *in vivo* validation is required. An *in vivo* model using BALB/c mice infected with S. Enteritidis was established, and a continuous observation with three selected time points was designed to better evaluate the virulence of SaaS compared with the single time point of similar researches (15, 38, 39). Although the incubation period (from infection to onset of symptoms) of Salmonella is 4 to 8 h, changes in the ethophysiology induced by sRNA SaaS were observed during 24 to 36 h, confirming the rationality of the time points in this study. The more rapid and frequently observed pathological phenomena and higher LD_50_ in Δ*saaS* strain-infected mice indicate that sRNA SaaS can be identified as an essential regulatory factor that positively regulates the virulence of S. Enteritidis. Moreover, compared with similar studies of sRNA virulence, the lower LD_50_ of this strain and the higher ratio of the LD_50_ between WT and Δ*saaS* strains indicate stronger pathogenicity of strain S. Enteritidis strain NMC61 and its sRNA SaaS (18). For orally infected mice, bacterial spread and colonization abilities in host organs are often deemed critical events in systemic S. Enteritidis pathogenesis (40). The lower number of viable bacteria of the Δ*saaS* strain recovered from the liver and intestine samples suggests that SaaS contributes to S. Enteritidis as a highly resilient bacterium suitable for various organ backgrounds, which is consistent with the results of LD_50_. For the spleen, where host defense limits bacterial replication and eliminates pathogens, the immune response containing most B cells and T cells may be an important reason for the failure of SaaS regulation. Compared with reported sRNAs that regulate virulence, such as DsrA, SaaS presented a stronger regulation of colonization in more tissues (39).

Inflammation is a physiological phenomenon that is activated by exogenous challenges, such as bacteria, viruses, and parasites, leading to tissue damage. Cytokines are important molecular markers for inflammation. IL-1β, IL-8, and TNF-α are typical proinflammatory factors that are downstream molecules of the NF-κB and p38/Jun N-terminal protein kinase (JNK) mitogen-activated protein kinase (MAPK) signaling pathways (41, 42). In this study, the higher levels of cytokines in WT-infected mice indicated a stronger acute inflammation, in keeping with the results of the survival rate in a mouse model and the bacterial burden in organs, which is also confirmed by similar research (43). Considering the strong ability to stimulate the host to produce endogenous cell factors, the higher levels of LPS in the WT group may be the reason for higher levels of IL-1β and TNF-α. A rapid and well-coordinated innate immune response is the first line of defense against infection; however, a maladjusted hyperimmune response can lead to severe organ damage, posing a self-perpetuating vicious cycle peaking at cytokine storm and death (44). Furthermore, inflammation can provide a growth advantage for Salmonella and enhance its systemic spread in animals (44, 45). As a result, the effect of SaaS on increasing cytokine secretion and decreasing host defense may lead to S. Enteritidis dissemination, such as the more extensive spread of S. Enteritidis to the liver.

### Conclusion.

This study reveals that SaaS is a critical sRNA that regulates the virulence of S. Enteritidis. To achieve this, SaaS was first found to be activated and to strengthen the modulation of virulence genes in SIE, with *ssaV* as a direct target. Furthermore, SaaS promotes the invasion of epithelial cells and decreases intracellular growth in macrophages, which is likely to be the cause of the increased intestinal barrier dysfunction and immune escape. More importantly, this study has highlighted the role of virulence in complex host environments in which the sRNA SaaS is required for higher lethality, further deterioration of ethophysiology, bacterial dissemination into systemic circulation, and systematic inflammation. Our research provides new paradigms for interactions between bacteria and the host immune response, which would further raise public concern about S. Enteritidis hazards, provide new targets for controlling S. Enteritidis in food, and reduce the risk of Salmonella infection.

## MATERIALS AND METHODS

### Ethics statement.

All experiments were carried out in compliance with the relevant guidelines and regulations of the Ethical Committee of the Experimental Animal Center of Nanjing Agricultural University.

### Bacterial cultures and growth conditions.

For the wild-type (WT) strain (46), S. Enteritidis strain NCM61 (reference genome CP032851.1), isolated from meat-contact surfaces and known to form biofilms on several surfaces (47, 48), was used. Biofilms of S. Enteritidis NCM61 previously demonstrated strong resistance to sanitizer acidic electrolyzed water. Additionally, the planktonic cells displayed strong tolerance to oxidative stress, similar to Salmonella ATCC 13076 with the Enteritidis serotype, particularly the highest tolerance to acidic and hyperosmotic stress in five tested Salmonella strains (14, 49). The test strain was stored at −80°C in Luria-Bertani broth (LB; Hope Biotechnology, Beijing, China) containing 60% (vol/vol) glycerol. It was streaked and grown twice overnight on LB agar for single colony isolation following incubation in 6 mL of fresh LB broth at 37°C for 20 h. The cells were harvested by centrifugation at 8,000 × *g* for 5 min at 4°C and washed three times with 0.85% NaCl solution. The pellets were then resuspended in an 0.85% NaCl solution to a final concentration of approximately 10^9^ CFU/mL, and the optical density value was measured at 600 nm (OD_600_) for subsequent experiments.

### Construction of the *saaS* deletion mutant and complemented strain.

The *saaS* deletion mutant was constructed through allelic exchange using the suicide plasmid. The upstream and downstream homologous recombination arms of the *saaS* gene from the WT genome and the kanamycin resistance (Kn^r^) gene from the pKD4 plasmid were amplified using the primer pairs *saaS*-5F/5R, *saaS*-3F/3R, and Kn-F/R, respectively (Table 1). The three sequences were bridged by fusion PCR to obtain the complete target fragment Δ*saaS*::Kn (upstream homologous arm-Kn^r^ gene-downstream homologous arm), and Δ*saaS*::Kn was then cloned into the general vector pUC19 to obtain the intermediate plasmid pUC19-Δ*saaS*::Kn. Second, the target fragment Δ*saaS*::Kn was subcloned into the suicide plasmid pCVD442 following confirmation by sequencing with the above intermediate plasmid to obtain pCVD442-Δ*saaS*::Kn. The donor strain β2155/pCVD442-Δ*saaS*::Kn was obtained by transferring pCVD442-Δ*saaS*::Kn into Escherichia coli β2155. The donor strain and the recipient strain WT were conjugated, and the Salmonella clones obtaining Kn^r^ were collected and named Sen/pCVD442-Δ*saaS*::Kn. Ultimately, knockout mutants (Δ*saaS*) were produced by electroporating plasmid pCP20 into the competent cells of Sen/pCVD442-Δ*saaS*::Kn and named S. Enteritidis strain NCM282.

**TABLE 1:** List of plasmids and primers used for strain construction in this study^*a*^

Primer or plasmid	Sequence (5′–3′) or characteristic	Description
Primers		
*saaS*-5F	ATAGTCGACTTCTATACGCCTGACTTTCCT	Upstream flanking region cloning
*saaS*-5R	TTAATGGTTCGCCATTTTTATGAATG	
*saaS*-3F	GGCTATTCAATATCATTGTTCTGTC	Downstream flanking region cloning
*saaS*-3R	ATAGTCGACACTAAATGACCATTCGTTGAAAG	
Kn-F	GACATTCATAAAAATGGCGAACCGAGCTGCTTCGAAGTTCCTA	Kn^r^ cloning
Kn-R	GACAGAACAATGATATTGAATAGCCCATATGAATATCCTCCTTAGTTCCTATTC	
*saaS*-outF2	GAGATACCGACGCAACGACTTG	Conjugation verification
*saaS*-outR2	CTTCATGGTTAATGGTCTGATAATTACACCATC	
*saaS*-outF	CAGAAGAAGGTACCAGCTTTAGCGAC	Knockout verification sequencing
pUC19-F	GTGCTGCAAGGCGATTAAGTT	Universal vector sequencing
pUC19-R	GCTCGTATGTTGTGTGGAATTG	
pRK415-F	CAACGCAATTAATGTGAGTTAGCTCAC	*saaS* cloning (vector segment)
pRK415-R	CTCTTCGCTATTACGCCAGCTG	*saaS* cloning (vector segment)
*saaS*-F	ATAAAGCTTGCATTAACATTTTTTGTATCTGTACTTAAG	*saaS* cloning
*saaS*-R	ATAGAATTCTCCCATCCTGATAGAGCG	*saaS* cloning
Plasmids		
pKD4	Kn^r^-containing plasmid	Kn^r^ cloning
pUC19	Universal sequencing plasmid	Target fragment verification
pCVD442	Suicide plasmid	Target fragment conjugative transfer
pCP20	Temperature-sensitive plasmid	Kn^r^ elimination
pRK415	Complemented plasmid	*saaS* complement

a Kn^r^, kanamycin resistance.

The *saaS* gene and the low-copy-number pRK415 expression vector were amplified for the complemented strain using primers *saaS*-F/R and pRK415-F/R, respectively. Following gel purification and HindIII/EcoRI digestion, the digested *saaS* and pRK415 products were ligated for 2 h at 37°C to generate the constructed plasmid pRK415-*saaS*. pRK415-*saaS* was subsequently transferred into the mutant Δ*saaS* strain and selected on tetracycline (Tc; 10 μg/mL) plates to yield the complemented strain (Δ*saaS*/p*saaS*).

### Bacterial growth curve assays.

The growth curves of WT, Δ*saaS*, and Δ*saaS*/p*saaS* strains in normal LB broth and SIE (0.3% bile salts and pH 6.8) were determined by incubating the strains for 48 h at 37°C. Sterile LB was used as a blank. The initial inoculum concentration was adjusted to approximately 10^2^ CFU/mL with sterile LB; the OD_600_ value was measured at 30-min intervals using a multimode microplate reader (SpectraMax M2e; Molecular Devices, San Jose, CA, USA). To validate the growth curves, aliquots of the bacterial cultures incubated for 12 h were collected for plating the cells on LB plates and counting the viable cells. Bacterial cultures in the exponential phase were collected to study the long-term effect of a possible *in vivo* environment on the expression of *saaS* and virulence-associated target genes.

### Expression of sRNA SaaS and virulence-associated target genes.

The virulence-associated target mRNAs of sRNA SaaS against the entire genome of S. Enteritidis NMC61 were predicted using the professional program IntaRNA (50). The information on potential RNA-RNA interactions is listed in Table S1 and Fig. S1 in the supplemental material. Total RNA was extracted from S. Enteritidis cells using a simple total RNA extraction kit (Tiangen, Beijing, China) following the manufacturer’s protocol. cDNA was synthesized using a PrimeScript reverse transcription (RT) reagent kit (TaKaRa, Dalian, Liaoning, China) at 37°C/15 min and 85°C/5 s according to the manufacturer’s recommendations. Subsequently, the expression levels of the target genes (*invA*, *spiA*, *prgJ*, *phoP*, *tssG*, *ssaV*, *ssaQ*, *ssaR*, *ssaT*, and *ssaU*) and sRNA SaaS were determined by reverse transcription coupled with quantitative real-time PCR (RT-qPCR). The following reagents were used in the RT-qPCR assays (20-μL final volume per sample): 10 μL of 2× SYBR Premix Ex SYBR (TaKaRa), 0.4 μL of each primer (10 mM), 0.4 μL of 50× ROX reference dye II, 2 μL of cDNA template (400 ng of total RNA), and 6.8 μL of RNase-free water. In parallel, 16S rRNA was used as an internal control for the normalization of gene expression data. The following protocol was used on a QuantStudio 6 Flex system (Applied Biosystems, Foster City, CA, USA): initial denaturation at 95°C/30 s; 40 cycles of denaturation at 95°C/5 s and annealing at 60°C/34 s; and a final melting curve program of 15 s at 95°C, 1 min at 60°C, and 15 s at 95°C. Differentially expressed target genes were calculated using the threshold cycle (2^−ΔΔ^*^CT^*) method. The primers used in the RT-qPCR assay are listed in Table S1.

### RNA-RNA pulldown assay.

The pulldown assay was performed as described previously (51). The biotinylated RNA probe targeting *ssaV* was generated using the Angle gene (Nanjing, Jiangsu, China) and incubated with streptavidin magnetic beads (Thermo Fisher Scientific, Waltham, MA, USA) at 4°C overnight. Beads were then collected and washed with ice-cold PBS. Subsequently, the beads that were combined with the biotinylated RNA probe were incubated for 1 h at 4°C with 60 μg of total RNA extracted from S. Enteritidis WT. Beads were collected and washed again with ice-cold PBS. Ultimately, the RNA bound to SaaS was eluted and subjected to RT-qPCR to examine the expression of *ssaV*.

### Western blot assays.

Polyclonal antibodies to SsaV were generated as described previously (52) by repeated immunization of New Zealand White rabbits with 1 mg of recombinant glutathione *S*-transferase (GST) fusion of SsaV. The GST fusion was constructed by PCR amplification of the *ssaV* gene from the genomic sequence of the WT using the following primers: forward, 5′-GCA GGA TCC ATT AAC ATT TTT TGT ATC TGT CAC TTA-3′, and reverse, 5′-CGT GTC GAC TCC CAT CCT GAT AGA GCG TG-3′. After digestion with BamHI and SalI, the resulting PCR product was cloned into pGEX6P-1. This plasmid was transformed into BL21 competent E. coli, and the production of recombinant fusion proteins was induced with 1 mM isopropyl-1-thio-beta-d-galactopyranoside. SsaV was purified using glutathione MagBeads (GenScript, Nanjing, Jiangsu, China), according to protocol. Raw antisera were affinity purified using protein A/G Sepharose beads (Thermo Fisher Scientific).

Western blotting was performed as described previously (53) with the following modifications: WT, Δ*saaS*, and Δ*saaS*/p*saaS* strains that were incubated in the SIE were harvested in the exponential phase and washed twice in ice-cold PBS. Cells were lysed in a protein extraction buffer containing protease and phosphatase inhibitors (Beyotime, Nantong, Jiangsu, China). Sixty micrograms of total protein was separated on a 10% SDS-PAGE gel at 100 V for 2 h and transferred to a polyvinylidene difluoride (PVDF) membrane (Millipore, Bedford, MA, USA). The membranes were probed with anti-SsaV antibodies at a dilution of 1:1,000. Commercial goat anti-mouse IgG(H+L) and goat anti-rabbit IgG(H+L) secondary antibodies (Thermo Fisher Scientific; 1:5,000) were used. Immunoblot signals were detected using the SuperSignal West Dura extended-duration substrate (Thermo Fisher Scientific).

### Cell experiments.

**(i) Bacterial adherence and invasion assays.** Bacterial intracellular behavior assays were performed as described for the epithelial cell line Caco-2 (54, 55) and macrophage cell line RAW 264.7 (54, 56). Caco-2 and RAW 264.7 cells were propagated in Dulbecco’s modified Eagle’s medium (DMEM) containing 10% heat-inactivated fetal bovine serum (FBS) and 1% antibiotics at 37°C in an atmosphere of 5% CO_2_. Prior to infection, the cells were washed three times with prewarmed PBS, and the medium was replaced with fresh DMEM without antibiotics and FBS.

For Caco-2 cells, a monolayer of cells was infected with an exponential-phase bacterial culture (multiplicity of infection [MOI] = 10) and incubated for 3 h (*t* = 0) at 37°C in a 5% CO_2_ atmosphere. Following incubation, the wells were washed six times with PBS, and 100 μg/mL gentamicin was added for 30 min to kill residual extracellular bacteria. Thereafter, fresh medium containing low-dose gentamicin (10 μg/mL) was added, and the cells were incubated for various intervals over a 24-h period. The cells were disrupted with 1% Triton X-100 at 37°C for 5 min at *t* = 0 (invasion), *t* = 1 (internalization), and rest intervals (replication). Lysate dilutions were plated on xylose lysine desoxycholate (XLD) agar, and the proportion of bacteria was determined by comparing the bacterial recovery from the initial inoculum.

For RAW 264.7 cells, the procedure was similar to that of Caco-2 cells, with an initial incubation of 1 h instead of 3 h. At the indicated time points, cells were lysed with 1% Triton X-100, followed by serial dilution on agar plates to quantify the intracellular bacterial burden.

**(ii) Cell cytotoxicity assay.** To verify the viability of Caco-2 cells and RAW 264.7 cells during different challenge times, the release of LDH was determined according to the instructions of the lactate dehydrogenase assay kit (Jiancheng, Nanjing, Jiangsu, China). The supernatants from the abovementioned tests were collected and frozen in microtubes at −80°C. Next, 20 μL of the supernatant was transferred to 96-well polystyrene plates. Subsequently, 30 μL of the reaction mixture from the LDH detection kit was added and incubated for 15 min at 37°C, followed by the addition of 25 μL of 2,4-dinitrophenylhydrazine and another 15-min incubation. Finally, 250 μL of 0.4 mol/L NaOH was transferred into plates. The contents were briefly mixed on an orbital shaker and incubated for 5 min at room temperature, followed by measurement using a multimode microplate reader at 450 nm. Relative LDH release was calculated as LDH release in the supernatant/(LDH release in the supernatant + LDH release in the cell fraction) × 100%.

### Animal experiments.

**(i) LD_50_**. Female BALB/c mice (6 to 8 weeks old) were maintained on a normal diet (standard mouse feed and sterile water) provided *ad libitum* for 7 days under specific-pathogen-free conditions. The temperature (22.0 ± 0.5°C) and relative humidity (60% ± 10%) were kept constant during the experiment, with a 12-h light cycle. The virulence of WT, Δ*saaS*, and Δ*saaS*/p*saaS* strains was evaluated in groups of 10 BALB/c mice by oral inoculation with various doses (10^3^ to 10^8^ CFU) of the bacterial strains as described previously (57). Water and food were withdrawn 4 h before oral gavage with 100 μL of 5% sodium bicarbonate to neutralize stomach acid, and 1 h later, mice were administered 10-fold dilutions of the WT, Δ*saaS*, or Δ*saaS*/p*saaS* strain in 100 μL of phosphate-buffered saline (PBS) by oral gavage. Control mice received 100 μL of PBS through the same route. The survival of mice was monitored, and the survival rate was recorded over the 14-day experimental period. The LD_50_ was calculated using the previously described protocol (58).

**(ii) Bacterial burden measurement.** Groups of 10 mice were orally infected by pipette feeding of 100 μL of PBS containing 10^8^ CFU of bacteria (WT, Δ*saaS*, or Δ*saaS*/p*saaS*) growing in the logarithmic phase and allowed access to water and food *ad libitum* for 5 days. The body weight and feed intake of the mice were routinely recorded. The infected mice were euthanized via cervical dislocation at 6, 72, and 120 h after infection. The liver, spleen, small intestine, and large intestine were collected, weighed, and homogenized in 0.3% Triton X-100 (Sigma-Aldrich, St. Louis, MO, USA). The homogenates were subsequently diluted, and the samples were plated on XLD to determine the number of CFU per gram of organ tissue.

**(iii) Organ coefficients.** The relative weights of the spleen and liver tissues were calculated according to their corresponding body weight.

**(iv) Inflammation levels.** Blood samples were collected, held at room temperature for 45 min, and then centrifuged at 3,000 × *g* for 30 min to pellet the blood cells. Serum samples were collected to assess secretion of cytokine, including interleukin-1β (IL-1β), interleukin-8 (IL-8), tumor necrosis factor alpha (TNF-α), and another lipopolysaccharide (LPS) using ELISA kits (Angle Gene, Nanjing, Jiangsu, China) according to the manufacturer’s instructions.

### Statistical analysis.

SAS software (version 9.2; SAS Institute Inc., Cary, NC, USA) was used for statistical analysis. Survival rates were compared using the log rank test. Differences between two groups were evaluated using an independent-sample *t* test. *P* values of ≤0.05 or 0.01 were considered to be statistically significant (*) and highly significant (**), respectively. All the data are representative of at least three independent experiments. Data are expressed as means ± standard deviations (SD), and figures were constructed using GraphPad Prism (version 5.0.3; GraphPad Software Inc., San Diego, CA, USA).
