# Supplementary material for: A Small RNA, SaaS, Promotes Salmonella Pathogenicity by Regulating Invasion, Intracellular Growth, and Virulence Factors
Source: Microbiol Spectr. 2023 Jan 23;11(1):e02938-22. doi: 10.1128/spectrum.02938-22 (PMC9927236; doi:10.1128/spectrum.02938-22)
Supplement: Supplemental file 1 — Fig. S1 and Table S1. Download spectrum.02938-22-s0001.pdf, PDF file, 0.5 MB [file spectrum.02938-22-s0001.pdf]

A small RNA SaaS promotes *Salmonella* pathogenicity by regulating invasion, intracellular growth and virulence factors

Lin-Lin Cai, Yun-Ting Xie, Hai-Jing Hu, Xing-Lian Xu*, Hu-Hu Wang* and Guang-Hong Zhou

Jiangsu Collaborative Innovation Center of Meat Production and Processing, Quality and Safety Control, Nanjing Agricultural University, Nanjing 210095, P.R. China

Corresponding author, E-mail address: xlxus@njau.edu.cn (Xing-lian Xu) and huuwang@njau.edu.cn (Hu-hu Wang).

Supplementary figure captions

Fig S1. Predicted SaaS and target mRNA interaction using IntaRNA.

Table S1 Predicted virulence associated target mRNA of sRNA SaaS and primers used for RT-qPCR analysis

Gene	Energy (kcal/mol)	Description	Primer (5'-3')	Product (bp)	Reference
invA	-9.39	EscV/YscV/HrcV family type III secretion system export apparatus protein	F: GCCTG CCGGAAGTATTGTTA<br>R: GGAGTTTCTCCCCC TCTTCA	222 bp	This study
spiA	-7.31	EscC/YscC/HrcC family type III secretion system outer membrane ring protein	F: ACTTCACCCTTTATGCCAGAC<br>R: GCCATCAAACCAGGTAAGC	191 bp	This study
prgJ	-5.44	EscF/YscF/HrpA family type III secretion system needle major subunit	F: TTGTCCCTGAGAATGCCG<br>R: ACAGCCCCGACTCCTTTAC	268 bp	This study
phoP	-5.29	Response regulator in two-component regulatory system with *phoQ*	F: GACGAAGCCATTCCACATC<br>R: AATCTTTGCTGACCACTTTACC	221 bp	This study
tssG	-9.47	Type VI secretion-associated protein, ImpA family	F: CGTTGCTGTAGGTGTAGTCGG<br>R: TTGCTCCCCATCCTTATCG	214 bp	This study
ssaV	-6.91	Secretion system apparatus protein	F: CGTAGGAGGAAATCTCACCG<br>R: GCCTGGCATCCCATCAA	113 bp	This study
ssaQ	-9.37	Type III secretion system protein	F: CATTGCTTCGGCGACATCAG<br>R: CGACCTCAAAGAGCACCTGT	217 bp	This study
ssaR	-6.9	EscR/YscR/HrcR family type III secretion system export apparatus protein	F: GTTTCTGCTTTCAATACTGCCTCT<br>R: CGCTAATGCTTTACTGTCCCAC	256 bp	This study
ssaT	-5.06	Type III secretion system protein	F: GATATGGCGGGGTTTCTGCT<br>R: CTGGCTGAAAAGCAAGCCAA	108 bp	This study
ssaU	-6.86	EscU/YscU/HrcU family type III secretion system export apparatus switch protein	F: GGGCGACCCTCAAATGAAGA<br>R: AGCTTGAGCATCACTGCCTT	190 bp	This study
16S rRNA		Reference gene	F: CGGGGAGGAAGGTGTTGTG<br>R: GAGCCCGGGGATTTCACATC	178 bp	This study

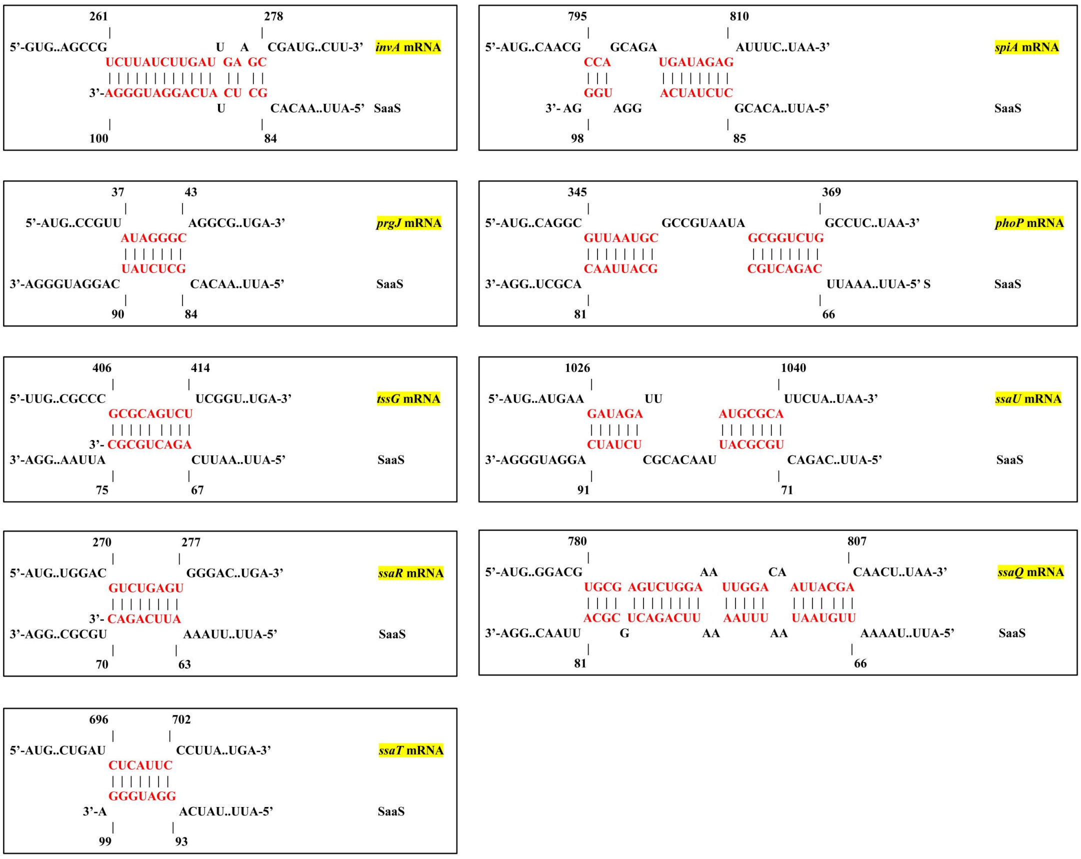

261 278
5’-GUG..AGCCG U A CGAUG..CUU-3’ invA mRNA
UCUUAUCUUGAU GA GC
3’-AGGGUAGGACUA CU CG
U CACAA..UUA-5’ SaaS
100 84
795 810
5’-AUG..CAACG GCAGA AUUUC..UAA-3’ spiA mRNA
CCA UGAUAGAG
GGU ACUAUCUC
3’- AG AGG GCACA..UUA-5’ SaaS
98 85
37 43
5’-AUG..CCGUU AGGCG..UGA-3’ prgJ mRNA
AUAGGGC
UAUCUCG
3’-AGGGUAGGAC CACAA..UUA-5’ SaaS
90 84
345 369
5’-AUG..CAGGC GCCGUAAUA GCCUC..UAA-3’ phoP mRNA
GUUAAUGC GCGGUCUG
CAAUUACG CGUCAGAC
3’-AGG..UCGCA UUAAA..UUA-5’ S SaaS
81 66
406 414
5’-UUG..CGCCC UCGGU..UGA-3’ tssG mRNA
GCGCAGUCU
3’- CGCGUCAGA
3’-AGG..AAUUA CUUAA..UUA-5’ SaaS
75 67
1026 1040
5’-AUG..AUGAA UU UUCUA..UAA-3’ ssaU mRNA
GAUAGA AUGCGCA
CUAUCU UACGCGU
3’-AGGGUAGGA CGCACAAU CAGAC..UUA-5’ SaaS
91 71
270 277
5’-AUG..UGGAC GGGAC..UGA-3’ ssaR mRNA
GUCUGAGU
3’- CAGACUUA
3’-AGG..CGCGU AAAUU..UUA-5’ SaaS
70 63
780 807
5’-AUG..GGACG AA CA CAACU..UAA-3’ ssaQ mRNA
UGCG AGUCUGGA UUGGA AUUACGA
ACGC UCAGACUU AAUUU UAAUGUU
3’-AGG..CAAUU G AA AA AAAAU..UUA-5’ SaaS
81 66
696 702
5’-AUG..CUGAU CCUUA..UGA-3’ ssaT mRNA
CUCAUUC
GGGUAGG
3’-A ACUAU..UUA-5’ SaaS
99 93

Fig S1
